# Crystal structures of two alkaline earth (*M* = Ba and Sr) dimanganese(II) iron(III) tris­(orthophosphates)

**DOI:** 10.1107/S2056989017006120

**Published:** 2017-04-28

**Authors:** Ghaleb Alhakmi, Abderrazzak Assani, Mohamed Saadi, Lahcen El Ammari

**Affiliations:** a Laboratoire de Chimie du Solide Appliquée, Faculty of Sciences, Mohammed V University in Rabat, Avenue Ibn Battouta, BP 1014, Rabat, Morocco

**Keywords:** crystal structure, BaMn_2_Fe(PO_4_)_3_, SrMn_2_Fe(PO_4_)_3_, transition metal, phosphates, solid-state reaction synthesis

## Abstract

Two new orthophosphates, BaMn_2_Fe(PO_4_)_3_ [barium dimanganese(II) iron(III) tris­(orthophosphate)] and SrMn_2_Fe(PO_4_)_3_ [strontium dimanganese(II) iron(III) tris­(orthophosphate)], were synthesized by solid-state reactions. They are isotypic and crystallize in the ortho­rhom­bic system with space group type *Pbcn*. Their crystal structures comprise infinite zigzag chains of edge-sharing FeO_6_ octa­hedra (point group symmetry .2.) and Mn_2_O_10_ double octa­hedra running parallel to [001], linked by two types of PO_4_ tetra­hedra. The so-formed three-dimensional framework delineates channels running along [001], in which the alkaline earth cations (site symmetry .2.) are located within a neighbourhood of eight O atoms.

## Abstract 2

The two orthophosphates BaMn_2_Fe(PO_4_)_3_ and SrMn_2_Fe(PO_4_)_3_ are isotypic and resemble an alluaudite-like structure. The chains characterizing the alluaudite structure are built up from edge-sharing [MnO_6_] and [FeO_6_] octa­hedra linked by PO_4_ tetra­hedra.

## Chemical context   

Considerable attention has been devoted to the preparation of new inorganic materials with open-framework structures (Rao *et al.*, 2001 ▸; Bouzidi *et al.*, 2015 ▸) due to their structural diversity covering a wide range of chemical compositions (Zhou *et al.*, 2002 ▸). In particular, transition-metal-based open-framework phosphates represent a highly attractive class of materials in industrial processes. In fact, their special framework structures lead to inter­esting properties that depend not only on the inclusion guest in the pores, but also on the chosen transition metal (Durio *et al.*, 2002 ▸; López *et al.*, 2004 ▸; Férey *et al.*, 2005 ▸). Typical examples are ion-exchangers (Jignasa *et al.*, 2006 ▸; Kullberg & Clearfield, 1981 ▸) and compounds with special magnetic (Chouaibi *et al.*, 2001 ▸; Ferdov *et al.*, 2008 ▸) and catal­ytic properties (Weng *et al.*, 2009 ▸).

In this context, our group focuses on the synthesis and characterization of new transition-metal phosphates crystallizing either in alluaudite- (Moore, 1971 ▸) or α-CrPO_4_-type structures (Attfield *et al.*, 1988 ▸). In attempts to obtain new compounds belonging to the latter structure type, we have synthesized and structurally characterized several new phosphates, including those with oxidation states of both +II and +III for manganese. These compounds have the general formula *M*Mn^III^Mn_2_^II^(PO_4_)_3_ (*M* = Pb, Sr, Ba) (Alhakmi *et al.*, 2013*a* ▸,*b* ▸; Assani *et al.*, 2013 ▸) and adopt the α-CrPO_4_ structure type. Recently, the phosphates Na_2_Co_2_Fe(PO_4_)_3_ (Bouraima *et al.*, 2015 ▸) and Na_1.67_Zn_1.67_Fe_1.33_(PO_4_)_3_ (Khmiyas *et al.*, 2015 ▸) with an alluaudite-like structure were also reported. As a continuation in this regard, we have now extended our investigations to the quaternary system *M*O/MnO/Fe_2_O_3_/P_2_O_5_, where *M* is a divalent cation. The present work deals with the synthesis and the crystal structures of two new isotypic alkaline earth manganese iron phosphates, namely, BaMn_2_Fe(PO_4_)_3_ and SrMn_2_Fe(PO_4_)_3_. Their structures show a similarity with that of *AM*_4_(PO_4_)_3_ phosphates where *A* is a monovalent cation and *M* a divalent cation (Daidouh *et al.*, 1999 ▸; Assaaoudi *et al.*, 2006 ▸).

## Structural commentary   

**Figure 1:**
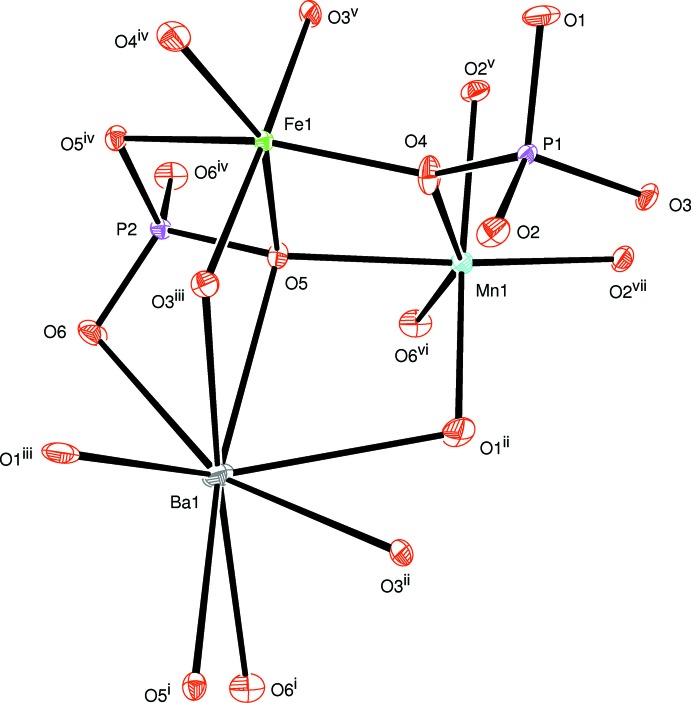
The principal building units in the structure of BaMn_2_Fe(PO_4_)_3_. Displacement ellipsoids are drawn at the 50% probability level. [Symmetry codes: (i) *x*, −*y* + 1, *z* + 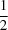; (ii) −*x* + 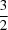, −*y* + 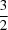, −*z* + 2; (iii) *x*, *y*, *z* + 1; (iv) −*x* + 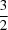, −*y* + 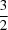, −*z* + 1; (v) −*x* + 1, *y*, −*z* + 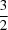; (vi) *x*, *y*, *z* − 1; (vii) −*x* + 1, *y*, −*z* + 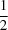; (viii) *x* − 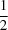, −*y* + 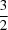, *z* − 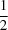; (ix) −*x* + 1, −*y* + 1, −*z* + 1; (x) *x*, −*y* + 1, *z* − 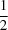; (xi) −*x* + 2, *y*, −*z* + 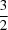; (xii) −*x* + 2, −*y* + 1, −*z* + 1.]

**Figure 3:**
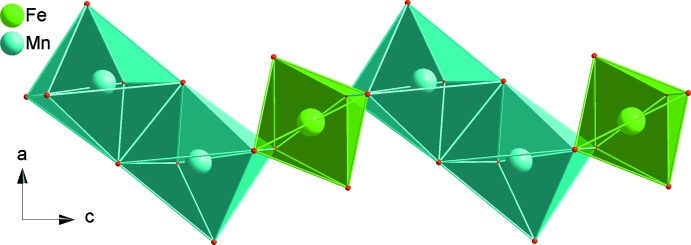
Edge-sharing [FeO_6_] octa­hedra and Mn_2_O_10_ dimers forming an infinite zigzag chain running parallel to [001]. Data are from BaMn_2_Fe(PO_4_)_3_.

**Figure 4:**
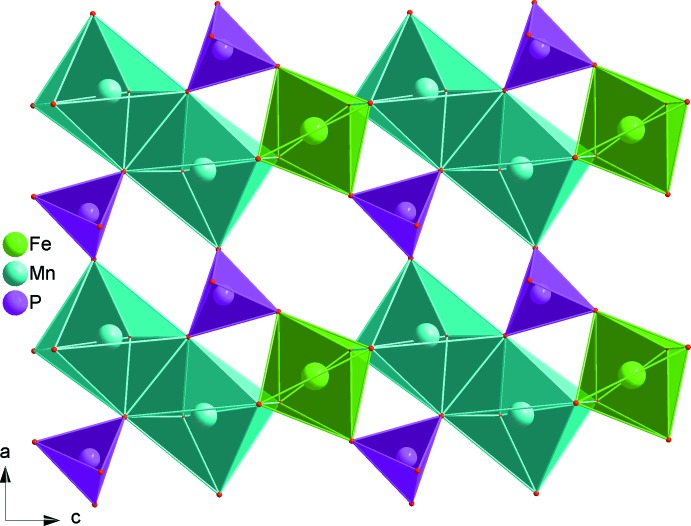
A layer perpendicular to (010), resulting from the connection of metal oxide chains through PO_4_ tetra­hedra. Data are from BaMn_2_Fe(PO_4_)_3_.

**Figure 5:**
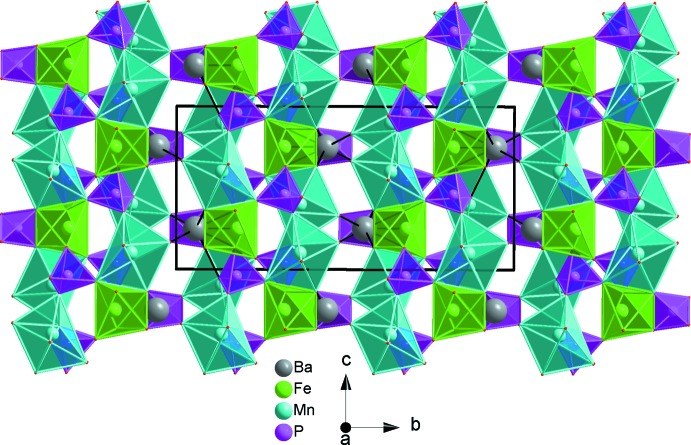
A view of stacked layers along [010]. Data are from BaMn_2_Fe(PO_4_)_3_.

The principal building units in the crystal structures of both phosphates are distorted FeO_6_ and MnO_6_ octa­hedra, PO_4_ tetra­hedra and Ba^2+^ or Sr^2+^ cations as shown in Figs. 1 ▸ and 2 ▸. In each structure, two MnO_6_ octa­hedra are linked together by a common edge to give a Mn_2_O_10_ dimer to which FeO_6_ octa­hedra (point group symmetry .2.) are alternately connected on both sides. In this way, infinite zigzag chains parallel to [001] are formed (Fig. 3 ▸). Adjacent chains are linked together by sharing corners with two types of PO_4_ tetra­hedra, forming a layer-like arrangement parallel to (010) as shown in Fig. 4 ▸. Such layers are stacked along [010] to form a three-dimensional framework (Fig. 5 ▸) with two types of channels running parallel to [001] in which the alkaline earth cations are located on a twofold rotation axis. They are coordinated by eight oxygen atoms (Figs. 1 ▸ and 6 ▸), with bond lengths ranging from 2.6803 (10) to 2.8722 (11) Å for the BaO_8_ polyhedron and of 2.6020 (9) to 2.7358 (11) Å for the SrO_8_ polyhedron.

Bond-valence-sum calculations (Brown & Altermatt, 1985 ▸) are in good agreement with the expected values for alkaline earth, manganese(II) and iron(III) cations and the phos­phorus(V) atom. BaMn_2_Fe(PO_4_)_3_ (values in valence units): Ba^2+^ 2.10; Mn^2+^ 2.00; Fe^3+^ 3.12; P^V^ 4.94. SrMn_2_Fe(PO_4_)_3_: Sr^2+^ 1.80; Mn^2+^ 2.07; Fe^3+^ 3.18; P^V^ 5.00.

## Database survey   

A comparison between the structures of the title compounds and those of other phosphates such as the *AM*_4_(PO_4_)_3_ compounds (*A* = monovalent cation and *M* = divalent cation) (Im *et al.*, 2014 ▸), reveals that all these compounds crystallize with ortho­rhom­bic symmetry and nearly the same unit-cell parameters despite the differences between their chemical formulae and space groups. In order to give an illustrative picture of the similarity between these two formula types, we can write the general formula of *AM*_4_(PO_4_)_3_ compounds as follows: *M*′^2+^(*A* ^+^ *M* ^2+^)*M*_2_^2+^(PO_4_)_3_ and that of the title compounds as *M*′^2+^Fe^3+^Mn_2_^2+^(PO_4_)_3_. The principal structures of the title compounds and that of the *AM*_4_(PO_4_)_3_ compounds are formed by stacking of the same infinite zigzag chains of edge-sharing octa­hedra. Furthermore, these structures are characterized by the presence of two types of channels in which the large cations are located.

## Synthesis and crystallization   

Single crystals of the title compounds were isolated as a result of solid-state reactions. Stoichiometric amounts of alkaline earth (*M* = Ba, Sr), manganese, iron and phosphate precursors in a molar ratio *M*:Mn:Fe:P = 1:2:1:3, were dissolved in 40 ml water that was placed into a 100 ml capacity pyrex glass beaker. The mixture was stirred at room temperature for 20 h and was evaporated under stirring at 363 K until dryness. The obtained black powder was ground in an agate mortar and pre-heated at 573 K in a platinum crucible for 24 h to eliminate gaseous materials. Subsequently, the resulting residue was reground and melted for 30 min at 1293 K, followed by slow cooling down to 1093 K at a rate 5K h^−1^ and a rapid cooling to room temperature by switching off the furnace. In the case of the BaO–MnO–Fe_2_O_3_–P_2_O_5_ system, the reaction product consisted of two types of crystals, *viz*. orange crystals of the title compound, BaMn_2_Fe(PO_4_)_3_, and dark-violet crystals that were identified to be another new phase. In the case of the SrO–MnO–Fe_2_O_3_–P_2_O_5_ system, the reaction product contained dark-brown crystals corresponding to the title compound, SrMn_2_Fe(PO_4_)_3_.

## Refinement   

Crystal data, data collection and structure refinement details for the two compounds are summarized in Table 1 ▸. For BaMn_2_Fe(PO_4_)_3_, the maximum and minimum remaining electron densities are located 0.60 and 0.42 Å from atom Ba1. For SrMn_2_Fe(PO_4_)_3_, they are 0.58 and 0.31 Å from Sr1.

## Supplementary Material

Crystal structure: contains datablock(s) I, II, global. DOI: 10.1107/S2056989017006120/wm5384sup1.cif

Structure factors: contains datablock(s) I. DOI: 10.1107/S2056989017006120/wm5384Isup2.hkl

Structure factors: contains datablock(s) II. DOI: 10.1107/S2056989017006120/wm5384IIsup3.hkl

CCDC references: 1545505, 1545504

Additional supporting information:  crystallographic information; 3D view; checkCIF report

## Figures and Tables

**Figure 2:**
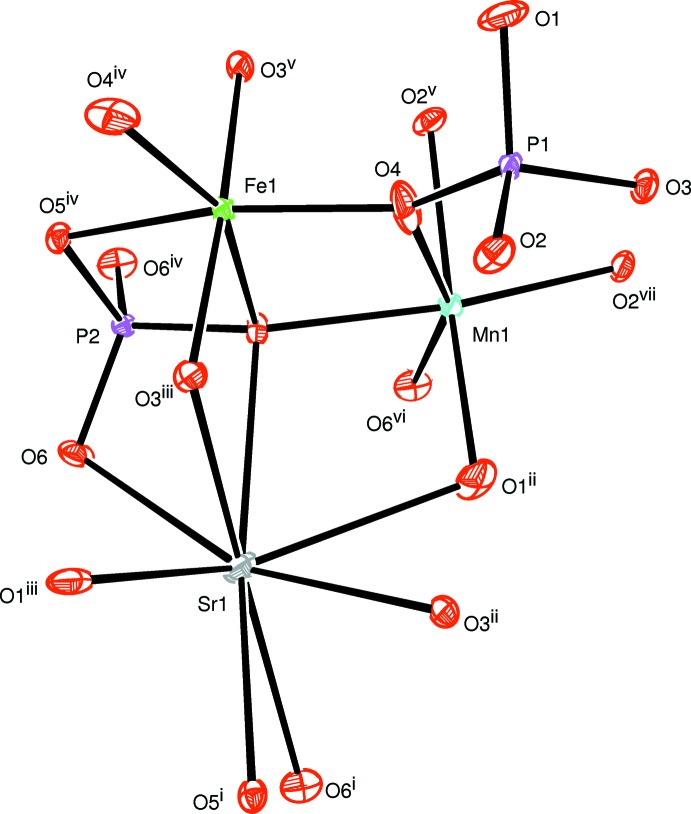
The principal building units in the structure of SrMn_2_Fe(PO_4_)_3_. Displacement ellipsoids are drawn at the 50% probability level. [Symmetry codes: (i) *x*, −*y* + 1, *z* + 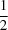; (ii) −*x* + 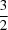, −*y* + 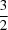, −*z* + 2; (iii) *x*, *y*, *z* + 1; (iv) −*x* + 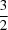, −*y* + 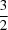, −*z* + 1; (v) −*x* + 1, *y*, −*z* + 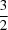; (vi) *x*, *y*, *z* − 1; (vii) −*x* + 1, *y*, −*z* + 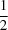; (viii) *x* − 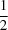, −*y* + 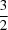, *z* − 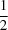; (ix) −*x* + 1, −*y* + 1, −*z* + 1; (x) *x*, −*y* + 1, *z* − 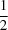; (xi) −*x* + 2, *y*, −*z* + 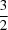; (xii) −*x* + 2, −*y* + 1, −*z* + 1.]

**Figure 6:**
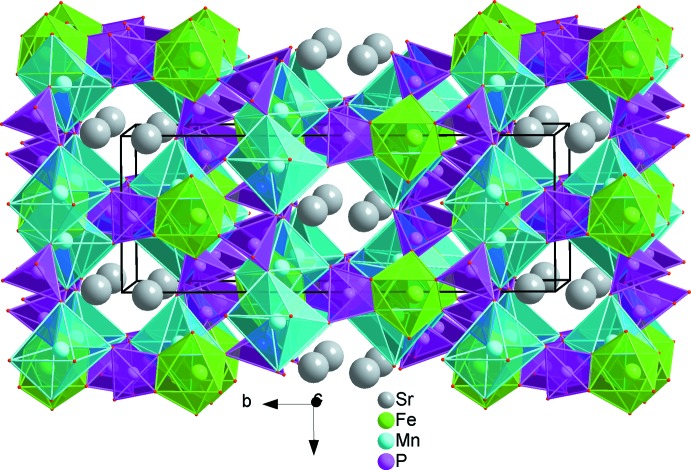
Polyhedral representation of the BaMn_2_Fe(PO_4_)_3_ structure showing Ba^2+^ cations situated in channels running along [001].

**Table 1:** Experimental details

	(I)	(II)
Crystal data		
Chemical formula	BaMn_2_Fe(PO_4_)_3_	SrMn_2_Fe(PO_4_)_3_
*M*_r_	587.98	538.25
Crystal system, space group	Orthorhombic, *P* *b* *c* *n*	Orthorhombic, *P* *b* *c* *n*
Temperature (K)	296	296
*a*, *b*, *c* (Å)	6.5899 (2), 17.6467 (4), 8.5106 (2)	6.4304 (3), 17.8462 (7), 8.4906 (3)
*V* (Å^3^)	989.70 (4)	974.37 (7)
*Z*	4	4
Radiation type	Mo *K*α	Mo *K*α
μ (mm^−1^)	8.41	10.00
Crystal size (mm)	0.32 × 0.25 × 0.22	0.30 × 0.27 × 0.23

Data collection		
Diffractometer	Bruker X8 APEX	Bruker X8 APEX
Absorption correction	Multi-scan (*SADABS*; Krause *et al.*, 2015 ▸)	Multi-scan (*SADABS*; Krause *et al.*, 2015 ▸)
*T*_min_, *T*_max_	0.596, 0.748	0.404, 0.748
No. of measured, independent and observed [*I* > 2σ(*I*)] reflections	29422, 3088, 2731	23889, 2843, 2564
*R*_int_	0.033	0.031
(sin θ/λ)_max_ (Å^−1^)	0.907	0.887

Refinement		
*R*[*F* ^2^ > 2σ(*F* ^2^)], *wR*(*F* ^2^), *S*	0.018, 0.044, 1.05	0.021, 0.048, 1.08
No. of reflections	3088	2843
No. of parameters	89	89
Δρ_max_, Δρ_min_ (e Å^−3^)	1.29, −1.11	1.19, −0.81

Computer programs: *APEX2* and *SAINT* (Bruker, 2014 ▸), *SHELXT2014* (Sheldrick, 2015*a* ▸), *SHELXL2014* (Sheldrick, 2015*b* ▸), *ORTEP-3 for Windows* (Farrugia, 2012 ▸), *DIAMOND* (Brandenburg, 2006 ▸) and *publCIF* (Westrip, 2010 ▸).

## supplementary crystallographic information

### (I) Barium dimanganese(II) iron(III) tris(orthophosphate) Crystal data

**Table:**

BaMn_2_Fe(PO_4_)_3_	*D*_x_ = 3.946 Mg m^−^^3^
*M**_r_* = 587.98	Mo *K*α radiation, λ = 0.71073 Å
Orthorhombic, *P**b**c**n*	Cell parameters from 3088 reflections
*a* = 6.5899 (2) Å	θ = 3.3–40.1°
*b* = 17.6467 (4) Å	µ = 8.41 mm^−^^1^
*c* = 8.5106 (2) Å	*T* = 296 K
*V* = 989.70 (4) Å^3^	Block, orange
*Z* = 4	0.32 × 0.25 × 0.22 mm
*F*(000) = 1092	

### (I) Barium dimanganese(II) iron(III) tris(orthophosphate) Data collection

**Table:**

Bruker X8 APEX diffractometer	3088 independent reflections
Radiation source: fine-focus sealed tube	2731 reflections with *I* > 2σ(*I*)
Graphite monochromator	*R*_int_ = 0.033
φ and ω scans	θ_max_ = 40.1°, θ_min_ = 3.3°
Absorption correction: multi-scan (*SADABS*; Krause *et al.*, 2015)	*h* = −8→11
*T*_min_ = 0.596, *T*_max_ = 0.748	*k* = −31→32
29422 measured reflections	*l* = −15→15

### (I) Barium dimanganese(II) iron(III) tris(orthophosphate) Refinement

**Table:**

Refinement on *F*^2^	0 restraints
Least-squares matrix: full	*w* = 1/[σ^2^(*F*_o_^2^) + (0.0178*P*)^2^ + 1.2088*P*] where *P* = (*F*_o_^2^ + 2*F*_c_^2^)/3
*R*[*F*^2^ > 2σ(*F*^2^)] = 0.018	(Δ/σ)_max_ = 0.002
*wR*(*F*^2^) = 0.044	Δρ_max_ = 1.29 e Å^−^^3^
*S* = 1.05	Δρ_min_ = −1.11 e Å^−^^3^
3088 reflections	Extinction correction: SHELXL2014 (Sheldrick, 2015*b*), Fc^*^=kFc[1+0.001xFc^2^λ^3^/sin(2θ)]^-1/4^
89 parameters	Extinction coefficient: 0.00278 (15)

### (I) Barium dimanganese(II) iron(III) tris(orthophosphate) Special details

**Table:**

Geometry. All esds (except the esd in the dihedral angle between two l.s. planes) are estimated using the full covariance matrix. The cell esds are taken into account individually in the estimation of esds in distances, angles and torsion angles; correlations between esds in cell parameters are only used when they are defined by crystal symmetry. An approximate (isotropic) treatment of cell esds is used for estimating esds involving l.s. planes.

### (I) Barium dimanganese(II) iron(III) tris(orthophosphate) Fractional atomic coordinates and isotropic or equivalent isotropic displacement parameters (Å^2^)

**Table:**

	*x*	*y*	*z*	*U*_iso_*/*U*_eq_	
Ba1	0.5000	0.44269 (2)	0.7500	0.01037 (3)	
Fe1	1.0000	0.31799 (2)	0.7500	0.00461 (4)	
Mn1	0.83899 (3)	0.36570 (2)	0.39874 (2)	0.00647 (4)	
P1	0.83270 (5)	0.17935 (2)	0.53771 (3)	0.00490 (5)	
P2	1.0000	0.47123 (2)	0.7500	0.00513 (7)	
O1	1.01958 (15)	0.12822 (6)	0.55338 (13)	0.01186 (16)	
O2	0.66250 (15)	0.15480 (5)	0.64868 (11)	0.00865 (14)	
O3	0.76365 (15)	0.17592 (5)	0.36487 (10)	0.00794 (14)	
O4	0.88706 (16)	0.26335 (5)	0.57277 (11)	0.01031 (15)	
O5	0.89269 (15)	0.41422 (5)	0.63609 (10)	0.00656 (13)	
O6	0.83805 (16)	0.51729 (5)	0.83211 (12)	0.00988 (15)	

### (I) Barium dimanganese(II) iron(III) tris(orthophosphate) Atomic displacement parameters (Å^2^)

**Table:**

	*U*^11^	*U*^22^	*U*^33^	*U*^12^	*U*^13^	*U*^23^
Ba1	0.00562 (5)	0.01428 (5)	0.01119 (5)	0.000	−0.00082 (3)	0.000
Fe1	0.00537 (9)	0.00435 (8)	0.00411 (8)	0.000	0.00015 (7)	0.000
Mn1	0.00598 (7)	0.00785 (6)	0.00559 (7)	−0.00021 (5)	0.00041 (6)	0.00013 (5)
P1	0.00360 (11)	0.00666 (10)	0.00443 (10)	−0.00050 (9)	−0.00036 (9)	−0.00071 (8)
P2	0.00571 (17)	0.00355 (13)	0.00611 (15)	0.000	0.00000 (13)	0.000
O1	0.0058 (4)	0.0170 (4)	0.0128 (4)	0.0047 (3)	−0.0016 (3)	−0.0004 (3)
O2	0.0064 (3)	0.0126 (3)	0.0070 (3)	−0.0017 (3)	0.0007 (3)	0.0021 (3)
O3	0.0066 (4)	0.0127 (3)	0.0045 (3)	0.0002 (3)	−0.0018 (3)	−0.0017 (3)
O4	0.0136 (4)	0.0087 (3)	0.0086 (3)	−0.0047 (3)	−0.0002 (3)	−0.0028 (3)
O5	0.0084 (3)	0.0058 (3)	0.0055 (3)	−0.0007 (3)	−0.0009 (3)	−0.0003 (2)
O6	0.0091 (4)	0.0071 (3)	0.0134 (4)	0.0017 (3)	0.0011 (3)	−0.0031 (3)

### (I) Barium dimanganese(II) iron(III) tris(orthophosphate) Geometric parameters (Å, º)

**Table:**

Ba1—O6	2.6803 (10)	Mn1—O6^vi^	2.1413 (9)
Ba1—O6^i^	2.6803 (10)	Mn1—O1^ii^	2.1466 (10)
Ba1—O3^ii^	2.7861 (9)	Mn1—O2^vii^	2.1587 (9)
Ba1—O3^iii^	2.7861 (9)	Mn1—O2^v^	2.1997 (10)
Ba1—O5	2.8087 (10)	Mn1—O5	2.2223 (9)
Ba1—O5^i^	2.8087 (10)	Mn1—O4	2.3572 (10)
Ba1—O1^ii^	2.8722 (11)	P1—O2	1.5289 (10)
Ba1—O1^iii^	2.8722 (11)	P1—O1	1.5325 (10)
Fe1—O4	1.9387 (9)	P1—O3	1.5409 (9)
Fe1—O4^iv^	1.9387 (9)	P1—O4	1.5540 (9)
Fe1—O3^v^	1.9965 (9)	P2—O6	1.5126 (10)
Fe1—O3^iii^	1.9965 (9)	P2—O6^iv^	1.5126 (10)
Fe1—O5^iv^	2.0792 (9)	P2—O5	1.5659 (9)
Fe1—O5	2.0793 (9)	P2—O5^iv^	1.5660 (9)
			
O6—Ba1—O6^i^	121.17 (4)	O4^iv^—Fe1—O5^iv^	85.00 (4)
O6—Ba1—O3^ii^	157.68 (3)	O3^v^—Fe1—O5^iv^	83.59 (4)
O6^i^—Ba1—O3^ii^	79.21 (3)	O3^iii^—Fe1—O5^iv^	91.36 (4)
O6—Ba1—O3^iii^	79.21 (3)	O4—Fe1—O5	85.00 (4)
O6^i^—Ba1—O3^iii^	157.68 (3)	O4^iv^—Fe1—O5	154.09 (4)
O3^ii^—Ba1—O3^iii^	82.60 (4)	O3^v^—Fe1—O5	91.36 (4)
O6—Ba1—O5	53.99 (3)	O3^iii^—Fe1—O5	83.59 (4)
O6^i^—Ba1—O5	139.79 (3)	O5^iv^—Fe1—O5	70.50 (5)
O3^ii^—Ba1—O5	105.04 (3)	O6^vi^—Mn1—O1^ii^	89.96 (4)
O3^iii^—Ba1—O5	58.11 (3)	O6^vi^—Mn1—O2^vii^	84.29 (4)
O6—Ba1—O5^i^	139.79 (3)	O1^ii^—Mn1—O2^vii^	101.01 (4)
O6^i^—Ba1—O5^i^	53.99 (3)	O6^vi^—Mn1—O2^v^	96.48 (4)
O3^ii^—Ba1—O5^i^	58.11 (3)	O1^ii^—Mn1—O2^v^	173.39 (4)
O3^iii^—Ba1—O5^i^	105.04 (3)	O2^vii^—Mn1—O2^v^	78.23 (4)
O5—Ba1—O5^i^	159.39 (3)	O6^vi^—Mn1—O5	82.51 (4)
O6—Ba1—O1^ii^	114.27 (3)	O1^ii^—Mn1—O5	87.96 (4)
O6^i^—Ba1—O1^ii^	90.97 (3)	O2^vii^—Mn1—O5	164.03 (4)
O3^ii^—Ba1—O1^ii^	51.86 (3)	O2^v^—Mn1—O5	94.35 (4)
O3^iii^—Ba1—O1^ii^	87.88 (3)	O6^vi^—Mn1—O4	154.91 (4)
O5—Ba1—O1^ii^	64.56 (3)	O1^ii^—Mn1—O4	92.91 (4)
O5^i^—Ba1—O1^ii^	105.89 (3)	O2^vii^—Mn1—O4	119.45 (3)
O6—Ba1—O1^iii^	90.97 (3)	O2^v^—Mn1—O4	81.90 (4)
O6^i^—Ba1—O1^iii^	114.27 (3)	O5—Mn1—O4	72.70 (3)
O3^ii^—Ba1—O1^iii^	87.88 (3)	O2—P1—O1	111.65 (6)
O3^iii^—Ba1—O1^iii^	51.86 (3)	O2—P1—O3	111.22 (5)
O5—Ba1—O1^iii^	105.89 (3)	O1—P1—O3	107.29 (6)
O5^i^—Ba1—O1^iii^	64.55 (3)	O2—P1—O4	108.71 (5)
O1^ii^—Ba1—O1^iii^	128.34 (4)	O1—P1—O4	111.08 (6)
O4—Fe1—O4^iv^	120.35 (6)	O3—P1—O4	106.79 (5)
O4—Fe1—O3^v^	88.85 (4)	O6—P2—O6^iv^	115.00 (8)
O4^iv^—Fe1—O3^v^	94.22 (4)	O6—P2—O5	108.22 (5)
O4—Fe1—O3^iii^	94.22 (4)	O6^iv^—P2—O5	112.20 (5)
O4^iv^—Fe1—O3^iii^	88.85 (4)	O6—P2—O5^iv^	112.20 (5)
O3^v^—Fe1—O3^iii^	173.83 (5)	O6^iv^—P2—O5^iv^	108.22 (5)
O4—Fe1—O5^iv^	154.09 (4)	O5—P2—O5^iv^	100.05 (7)

Symmetry codes: (i) −*x*+1, *y*, −*z*+3/2; (ii) *x*−1/2, −*y*+1/2, −*z*+1; (iii) −*x*+3/2, −*y*+1/2, *z*+1/2; (iv) −*x*+2, *y*, −*z*+3/2; (v) *x*+1/2, −*y*+1/2, −*z*+1; (vi) *x*, −*y*+1, *z*−1/2; (vii) −*x*+3/2, −*y*+1/2, *z*−1/2.

### (II) Strontium dimanganese(II) iron(III) tris(orthophosphate) Crystal data

**Table:**

SrMn_2_Fe(PO_4_)_3_	*D*_x_ = 3.669 Mg m^−^^3^
*M**_r_* = 538.25	Mo *K*α radiation, λ = 0.71073 Å
Orthorhombic, *P**b**c**n*	Cell parameters from 2843 reflections
*a* = 6.4304 (3) Å	θ = 3.3–39.1°
*b* = 17.8462 (7) Å	µ = 10.00 mm^−^^1^
*c* = 8.4906 (3) Å	*T* = 296 K
*V* = 974.37 (7) Å^3^	Block, dark brown
*Z* = 4	0.30 × 0.27 × 0.23 mm
*F*(000) = 1020	

### (II) Strontium dimanganese(II) iron(III) tris(orthophosphate) Data collection

**Table:**

Bruker X8 APEX diffractometer	2843 independent reflections
Radiation source: fine-focus sealed tube	2564 reflections with *I* > 2σ(*I*)
Graphite monochromator	*R*_int_ = 0.031
φ and ω scans	θ_max_ = 39.1°, θ_min_ = 3.3°
Absorption correction: multi-scan (*SADABS*; Krause *et al.*, 2015)	*h* = −11→10
*T*_min_ = 0.404, *T*_max_ = 0.748	*k* = −31→31
23889 measured reflections	*l* = −8→15

### (II) Strontium dimanganese(II) iron(III) tris(orthophosphate) Refinement

**Table:**

Refinement on *F*^2^	0 restraints
Least-squares matrix: full	*w* = 1/[σ^2^(*F*_o_^2^) + (0.0183*P*)^2^ + 1.2279*P*] where *P* = (*F*_o_^2^ + 2*F*_c_^2^)/3
*R*[*F*^2^ > 2σ(*F*^2^)] = 0.021	(Δ/σ)_max_ = 0.001
*wR*(*F*^2^) = 0.048	Δρ_max_ = 1.19 e Å^−^^3^
*S* = 1.08	Δρ_min_ = −0.81 e Å^−^^3^
2843 reflections	Extinction correction: SHELXL2014 (Sheldrick, 2015*b*), Fc^*^=kFc[1+0.001xFc^2^λ^3^/sin(2θ)]^-1/4^
89 parameters	Extinction coefficient: 0.0072 (3)

### (II) Strontium dimanganese(II) iron(III) tris(orthophosphate) Special details

**Table:**

Geometry. All esds (except the esd in the dihedral angle between two l.s. planes) are estimated using the full covariance matrix. The cell esds are taken into account individually in the estimation of esds in distances, angles and torsion angles; correlations between esds in cell parameters are only used when they are defined by crystal symmetry. An approximate (isotropic) treatment of cell esds is used for estimating esds involving l.s. planes.

### (II) Strontium dimanganese(II) iron(III) tris(orthophosphate) Fractional atomic coordinates and isotropic or equivalent isotropic displacement parameters (Å^2^)

**Table:**

	*x*	*y*	*z*	*U*_iso_*/*U*_eq_	
Sr1	0.5000	0.43233 (2)	0.7500	0.00986 (4)	
Fe1	1.0000	0.31546 (2)	0.7500	0.00485 (4)	
Mn1	0.83818 (3)	0.37163 (2)	0.39547 (2)	0.00679 (4)	
P1	0.83555 (5)	0.17749 (2)	0.53581 (3)	0.00571 (5)	
P2	1.0000	0.46759 (2)	0.7500	0.00485 (7)	
O1	1.02378 (15)	0.12570 (6)	0.54770 (13)	0.01473 (18)	
O2	0.66091 (14)	0.15203 (5)	0.64550 (11)	0.00922 (14)	
O3	0.76936 (14)	0.17505 (5)	0.36165 (10)	0.00794 (14)	
O4	0.89115 (17)	0.25971 (6)	0.57468 (12)	0.01448 (18)	
O5	0.89256 (14)	0.41164 (5)	0.63388 (10)	0.00662 (13)	
O6	0.82775 (15)	0.51251 (5)	0.82684 (12)	0.00991 (14)	

### (II) Strontium dimanganese(II) iron(III) tris(orthophosphate) Atomic displacement parameters (Å^2^)

**Table:**

	*U*^11^	*U*^22^	*U*^33^	*U*^12^	*U*^13^	*U*^23^
Sr1	0.00601 (7)	0.01296 (7)	0.01060 (7)	0.000	−0.00134 (5)	0.000
Fe1	0.00567 (9)	0.00423 (8)	0.00466 (8)	0.000	0.00026 (7)	0.000
Mn1	0.00547 (7)	0.00927 (7)	0.00563 (7)	−0.00023 (5)	0.00064 (5)	−0.00056 (5)
P1	0.00382 (10)	0.00858 (11)	0.00473 (10)	−0.00077 (9)	−0.00010 (9)	−0.00167 (8)
P2	0.00502 (15)	0.00357 (13)	0.00597 (15)	0.000	−0.00017 (12)	0.000
O1	0.0066 (4)	0.0237 (5)	0.0139 (4)	0.0062 (3)	−0.0013 (3)	0.0010 (4)
O2	0.0061 (3)	0.0149 (4)	0.0067 (3)	−0.0015 (3)	0.0009 (3)	0.0025 (3)
O3	0.0067 (3)	0.0125 (3)	0.0046 (3)	0.0001 (3)	−0.0012 (3)	−0.0013 (3)
O4	0.0174 (4)	0.0133 (4)	0.0128 (4)	−0.0084 (3)	0.0029 (3)	−0.0074 (3)
O5	0.0085 (3)	0.0062 (3)	0.0052 (3)	−0.0009 (3)	−0.0016 (3)	−0.0006 (2)
O6	0.0085 (3)	0.0073 (3)	0.0140 (4)	0.0019 (3)	0.0015 (3)	−0.0032 (3)

### (II) Strontium dimanganese(II) iron(III) tris(orthophosphate) Geometric parameters (Å, º)

**Table:**

Sr1—O3^i^	2.6020 (9)	Mn1—O1^i^	2.0790 (10)
Sr1—O3^ii^	2.6020 (9)	Mn1—O2^v^	2.1462 (9)
Sr1—O6^iii^	2.6296 (9)	Mn1—O6^vi^	2.1494 (9)
Sr1—O6	2.6296 (10)	Mn1—O2^vii^	2.1641 (9)
Sr1—O5	2.7351 (9)	Mn1—O5	2.1748 (9)
Sr1—O5^iii^	2.7351 (9)	Mn1—O4	2.5338 (12)
Sr1—O1^i^	2.7358 (11)	P1—O1	1.5263 (10)
Sr1—O1^ii^	2.7358 (11)	P1—O2	1.5281 (9)
Fe1—O4	1.9224 (10)	P1—O3	1.5394 (9)
Fe1—O4^iv^	1.9224 (10)	P1—O4	1.5459 (10)
Fe1—O3^v^	1.9818 (9)	P2—O6	1.5149 (9)
Fe1—O3^ii^	1.9818 (9)	P2—O6^iv^	1.5149 (9)
Fe1—O5	2.0966 (9)	P2—O5^iv^	1.5641 (9)
Fe1—O5^iv^	2.0966 (9)	P2—O5	1.5641 (9)
			
O3^i^—Sr1—O3^ii^	85.14 (4)	O4^iv^—Fe1—O5	155.20 (4)
O3^i^—Sr1—O6^iii^	81.58 (3)	O3^v^—Fe1—O5	89.60 (4)
O3^ii^—Sr1—O6^iii^	161.43 (3)	O3^ii^—Fe1—O5	82.35 (4)
O3^i^—Sr1—O6	161.43 (3)	O4—Fe1—O5^iv^	155.20 (4)
O3^ii^—Sr1—O6	81.58 (3)	O4^iv^—Fe1—O5^iv^	86.54 (4)
O6^iii^—Sr1—O6	114.07 (4)	O3^v^—Fe1—O5^iv^	82.35 (4)
O3^i^—Sr1—O5	107.18 (3)	O3^ii^—Fe1—O5^iv^	89.60 (4)
O3^ii^—Sr1—O5	60.39 (3)	O5—Fe1—O5^iv^	70.09 (5)
O6^iii^—Sr1—O5	136.36 (3)	O1^i^—Mn1—O2^v^	169.25 (4)
O6—Sr1—O5	54.77 (3)	O1^i^—Mn1—O6^vi^	90.60 (4)
O3^i^—Sr1—O5^iii^	60.39 (3)	O2^v^—Mn1—O6^vi^	100.11 (4)
O3^ii^—Sr1—O5^iii^	107.18 (3)	O1^i^—Mn1—O2^vii^	103.58 (4)
O6^iii^—Sr1—O5^iii^	54.77 (3)	O2^v^—Mn1—O2^vii^	78.47 (4)
O6—Sr1—O5^iii^	136.36 (3)	O6^vi^—Mn1—O2^vii^	85.52 (4)
O5—Sr1—O5^iii^	164.49 (4)	O1^i^—Mn1—O5	86.15 (4)
O3^i^—Sr1—O1^i^	54.30 (3)	O2^v^—Mn1—O5	93.44 (4)
O3^ii^—Sr1—O1^i^	91.49 (3)	O6^vi^—Mn1—O5	86.65 (4)
O6^iii^—Sr1—O1^i^	91.23 (3)	O2^vii^—Mn1—O5	167.55 (4)
O6—Sr1—O1^i^	112.98 (3)	O1^i^—Mn1—O4	90.54 (4)
O5—Sr1—O1^i^	64.17 (3)	O2^v^—Mn1—O4	79.18 (4)
O5^iii^—Sr1—O1^i^	109.48 (3)	O6^vi^—Mn1—O4	157.72 (4)
O3^i^—Sr1—O1^ii^	91.49 (3)	O2^vii^—Mn1—O4	115.78 (3)
O3^ii^—Sr1—O1^ii^	54.30 (3)	O5—Mn1—O4	71.23 (3)
O6^iii^—Sr1—O1^ii^	112.98 (3)	O1—P1—O2	111.25 (6)
O6—Sr1—O1^ii^	91.23 (3)	O1—P1—O3	105.40 (6)
O5—Sr1—O1^ii^	109.48 (3)	O2—P1—O3	111.95 (5)
O5^iii^—Sr1—O1^ii^	64.17 (3)	O1—P1—O4	112.17 (6)
O1^i^—Sr1—O1^ii^	135.52 (5)	O2—P1—O4	108.80 (6)
O4—Fe1—O4^iv^	117.67 (7)	O3—P1—O4	107.21 (6)
O4—Fe1—O3^v^	89.54 (4)	O6—P2—O6^iv^	116.11 (7)
O4^iv^—Fe1—O3^v^	95.53 (4)	O6—P2—O5^iv^	112.91 (5)
O4—Fe1—O3^ii^	95.53 (4)	O6^iv^—P2—O5^iv^	106.63 (5)
O4^iv^—Fe1—O3^ii^	89.54 (4)	O6—P2—O5	106.64 (5)
O3^v^—Fe1—O3^ii^	170.19 (5)	O6^iv^—P2—O5	112.91 (5)
O4—Fe1—O5	86.54 (4)	O5^iv^—P2—O5	100.66 (7)

Symmetry codes: (i) *x*−1/2, −*y*+1/2, −*z*+1; (ii) −*x*+3/2, −*y*+1/2, *z*+1/2; (iii) −*x*+1, *y*, −*z*+3/2; (iv) −*x*+2, *y*, −*z*+3/2; (v) *x*+1/2, −*y*+1/2, −*z*+1; (vi) *x*, −*y*+1, *z*−1/2; (vii) −*x*+3/2, −*y*+1/2, *z*−1/2.
